# Active PI3K Pathway Causes an Invasive Phenotype Which Can Be Reversed or Promoted by Blocking the Pathway at Divergent Nodes

**DOI:** 10.1371/journal.pone.0036402

**Published:** 2012-05-03

**Authors:** Jeffrey J. Wallin, Jane Guan, Kyle A. Edgar, Wei Zhou, Ross Francis, Anthony C. Torres, Peter M. Haverty, Jeffrey Eastham-Anderson, Sabrina Arena, Alberto Bardelli, Sue Griffin, John E. Goodall, Kyla M. Grimshaw, Klaus P. Hoeflich, Christopher Torrance, Marcia Belvin, Lori S. Friedman

**Affiliations:** 1 Departments of Cancer Signaling and Translational Oncology, Genentech, Inc., South San Francisco, California, United States of America; 2 Bioinformatics, Genentech, Inc., South San Francisco, California, United States of America; 3 Pathology, Genentech, Inc., South San Francisco, California, United States of America; 4 Laboratory of Molecular Genetics, Institute for Cancer Research and Treatment, University of Torino Medical School, Candiolo, Italy; 5 FIRC Institute of Molecular Oncology, Milan, Italy; 6 Horizon Discovery Ltd., Cambridge, United Kingdom; University of Leuven (KU Leuven), Faculty of Medicine, Belgium

## Abstract

The PTEN/PI3K pathway is commonly mutated in cancer and therefore represents an attractive target for therapeutic intervention. To investigate the primary phenotypes mediated by increased pathway signaling in a clean, patient-relevant context, an activating PIK3CA mutation (H1047R) was knocked-in to an endogenous allele of the MCF10A non-tumorigenic human breast epithelial cell line. Introduction of an endogenously mutated PIK3CA allele resulted in a marked epithelial-mesenchymal transition (EMT) and invasive phenotype, compared to isogenic wild-type cells. The invasive phenotype was linked to enhanced PIP_3_ production via a S6K-IRS positive feedback mechanism. Moreover, potent and selective inhibitors of PI3K were highly effective in reversing this phenotype, which is optimally revealed in 3-dimensional cell culture. In contrast, inhibition of Akt or mTOR exacerbated the invasive phenotype. Our results suggest that invasion is a core phenotype mediated by increased PTEN/PI3K pathway activity and that therapeutic agents targeting different nodes of the PI3K pathway may have dramatic differences in their ability to reverse or promote cancer metastasis.

## Introduction

Genetic deviations in the phosphatidylinoisitol 3′-kinase (PI3K) pathway have been detected in many human cancers [1] and are thought to act primarily to stimulate cell proliferation and survival. Two hotspot mutations reside in the helical domain of p110α and a third is in the kinase domain. All three mutations have been shown to provide a gain of function for the PI3K enzyme, and can lead to increased downstream signaling through kinases such as Akt and mTOR [2], [3]. Genetic deletion or loss of function mutations within the tumor suppressor PTEN, a phosphatase with opposing function to PI3K, also increases PI3K pathway signaling [4]. In addition, activation of the PI3K pathway results in feedback down-regulation of pathway signaling, mediated by an mTOR/S6K phosphorylation and inhibition of IRS-1 at Ser612 [5], [6], [7].

Epithelial-mesenchymal transition (EMT) is a program of gene expression changes that leads to a dramatic shift in cell phenotype towards a more invasive and migratory behavior [8], [9]. The contribution of specific cancer mutations towards EMT is still not fully clear, and is confounded by the analysis of ectopic overexpression models that do not reflect the endogenous expression of mutated oncogenes. Studies in isogenic lines have shown that transgenic approaches can over-estimate the gain of function phenotypes induced by single cancer gene events especially effects on cell proliferation [10], [11].

In recent years gene targeting approaches have been utilized to introduce mutations into human cell lines in a disease relevant context [10], [11]. In these studies, ‘knock-in’ cell lines harboring an endogenous p110α kinase domain H1047R mutation were used to precisely evaluate the functional consequences of a PIK3CA mutation starting in a non-tumorigenic background. We found that PIK3CA mutations increase PTEN/PI3K pathway signaling and cell proliferation, but also promote EMT and cell invasion and these phenotypes are sensitive to potent and selective PI3K inhibitors. We also discovered that Akt or mTOR inhibition enhanced morphologies associated with PTEN/PI3K pathway signaling through feedback to PIP_3_.

## Materials and Methods

### Cell culture

Parental and knock-in MCF10A clones (H1047R A and B) were first published by Di Nicolantonio and colleagues [10] and were licensed from Horizon Discovery Ltd. An additional set of matched isogenic MCF10A parental PI3K mutant cells [12] were obtained from Horizon Discovery to confirm results of 3-D culture experiments. Cells were cultured in F12:DMEM 50∶50 medium supplemented with 20 ng/ml human EGF, 10 µg/ml insulin, 0.2 µg/ml hydrocortisone, 10% FBS, 100 units/ml penicillin, 2 mM L-glutamine, and 100 mg/ml streptomycin at 37°C under 5% CO_2_. MCF10A cells were typically passaged and maintained in the presence of EGF and insulin. To detect differences with the p110α H1047R and parental isogenic pairs, EGF and insulin were absent from the media, except for those studies associated with 3-D culture. Breast tumor cell lines were obtained from the American Type Culture Collection (ATCC). Cell lines were tested and authenticated using gene expression and single nucleotide polymorphism genotyping arrays, as previously described [13], [14]. Lines were cultured in RPMI or MEM supplemented with 10% fetal bovine serum, 100 units/ml penicillin, and 100 µg/ml streptomycin at 37°C under 5% CO_2_.

### Reagents

GDC-0941, PI3Ki-A/D, PI103 and erlotinib were from Genentech, Inc. mTOR1/2i is from patent WO 2008/023159 A1 [15]. AKT1/2i (Inhibitor VIII) was from EMD Chemicals. Human EGF, insulin, hydrocortisone and ®Actin antibodies were from Sigma. Antibodies to phospho-Akt^Thr308^, phospho-Akt^Ser473^, total Akt, Akt1, Akt2, phospho-PRAS40^Thr246^, PRAS40, phospho-S6^Ser235/236^, phospho-GSK3β^Ser9^, phospho-P70S6K^Thr389^, phospho-IRS1^Ser612^ and mTOR were from Cell Signaling. The p110α antibody was from BD Biosciences and the BrdU Proliferation ELISA was obtained from Roche.

### siRNAs and Transfections

mTOR and the non-targeting control siRNAs were from Dharmacon and the p110α siRNA (GGACAACTGTTTCATATAGTT) was from Genentech. The Akt siRNA inhibition kit, which inhibits the expression of both Akt1 and Akt2 was obtained from Cell Signaling. Transfection of siRNAs was accomplished using an Amaxa Nucleofector Kit as described by the manufacturer for MCF10A cells.

### Cell Viability Assays

Cells were seeded at 2000 cells/well in 384-well plates. Diluted compounds were added to quadruplicate wells in 384-well cell plates. After 4 days incubation, relative numbers of viable cells were measured using CellTiter-Glo (Promega) and read with a Wallac Multilabel Reader (PerkinElmer). EC_50_ values were calculated using Prism 4.0 (GraphPad).

### Protein Assays

Two million cells were seeded in a 10 cm^2^ tissue culture plate overnight. Cells were treated with an EC_50_ concentration of inhibitors for the times indicated. Following treatment, cells were washed with cold PBS and lysed in 1× Cell Extraction Buffer (Biosource) supplemented with protease inhibitors (Roche), 1 mM PMSF, and Phosphatase Inhibitor Cocktails 1 and 2 (Sigma). For immunoblots, equal protein amounts were separated by electrophoresis through NuPage Bis-Tris 10% gradient gels (Invitrogen); proteins were transferred onto PVDF membranes using the Criterion system from Bio-Rad.

### PIP_3_ Assay

The measurement of PIK3CA activity was carried out through a PIP_3_ Mass Strip Kit or a PI3K kinase ELISA assay (Echelon Biosciences) according to the manufacturer's instructions.

### BrdU Assay

Cells were seeded at 10,000 cells per well in a 96-well plate overnight. After 48 hours of culture with the BrdU reagent, the BrdU ELISA was performed according to the manufacturer's instructions (Roche) and the plate was read on an EnVision Plate Reader (PerkinElmer).

### Microarray Studies

Gene expression analysis was carried out on RNA extracted from sub-confluent cell cultures using Qiagen RNeasy kits. RNA samples of sufficient quality were profiled on Affymetrix HGU133Plus_2.0 chips. Preparation of complementary RNA, array hybridizations, scanning and subsequent array image data analysis were done using the manufacturer's specified protocol. Gene expression data for experiments described in this study has been deposited in the Gene Expression Omnibus (GEO) database under accession number GSE17785.

### 3-Dimensional Cell Culture and Reagents

Acinar growth and morphogenesis assays in 3D laminin-rich extracellular matrix (lrECM) were performed as described previously [16], [17]. Briefly, tissue culture dishes were coated with a thin layer of growth factor-reduced Matrigel (BD Biosciences) on ice and placed in a 37°C incubator for approximately 15 minutes to allow for matrix polymerization. Cells were dislodged with trypsin and resuspended in complete growth media containing EGF and insulin and supplemented with 5% Matrigel. After mixing, cells were added to thepre-coated plates in the presence or absence of inhibitors.

### Quantification of Acinar Size and Shape

Size and shape values were determined from multiple phase-contrast images as previously described [18]. An object's shape factor is defined as (4π*Area)/Perimeter^2^). This results in a value from 0 to 1, where 0 is a flat line and 1 is a perfect circle.

### Matrigel Invasion Assay

Migration through matrigel was performed using Biocoat Matrigel Invasion Chambers containing FluoroBlok inserts (BD Biosciences). Cells were plated (35,000 total) and allowed to migrate through matrigel for 24 hours according to the protocol provided by the manufacturer. Number of invaded cells was detected and quantified using ImageXpress (Molecular Devices).

## Results and Discussion

### PIK3CA kinase domain mutations and PI3K pathway signaling

**Figure 1:**
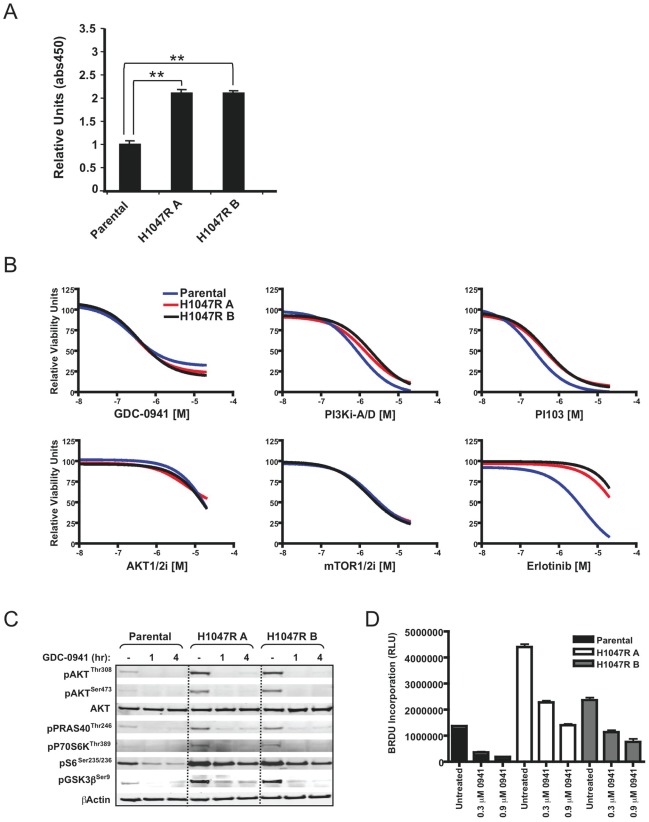
Effects of the H1047R mutation on PI3K pathway signaling, cell viability and proliferation. (A) Analysis of PIP_3_ levels in parental and knock-in clones. (B) Parental and knock-in clones were cultured with dose-titrated small molecule inhibitors and cell viability was assessed after four days. (C) Parental and knock-in clones were cultured in the presence or absence of GDC-0941 dosed at an EC_50_ concentration for the time points indicated and analyzed by Western blotting. (D) The parental and knock-in clones were cultured in the presence or absence of GDC-0941 for 48 hours and proliferation was assessed in triplicate samples by BrdU labeling.

A human mammary epithelial cell line, MCF10A, carrying an endogenous H1047R PI3K mutation “knocked in” to one allele [10] was used to investigate cellular changes associated with oncogenic PIK3CA. Two individual knock-in clones were utilized and both were found to have increased levels of phosphatidylinositol (3,4,5)-trisphosphate (PIP_3_), a product of PI3K enzymatic activity (Figure 1A).

We determined half maximal effective concentrations (EC_50_s) with small molecule inhibitors targeting PI3K, Akt, mTOR, and EGFR in the parental and knock-in clones in a growth inhibition format assay using CellTiter-Glo (Figure 1B and Table 1). Specificities and potencies for the compounds are shown in Table 1. GDC-0941 is a potent inhibitor of all class I PI3K isoforms, and is currently being evaluated in clinical trials [19]. PI3Ki-A/D is related to GDC-0941, but has reduced potency against the p110βclass I PI3K isoform [20]. PI103 is a pan class I PI3K isoform inhibitor and also displays significant potency against mTOR and DNA-PK [21], [22]. To evaluate the effects of inhibition downstream of PI3K, but still in the PI3K pathway, we utilized inhibitors of Akt and mTOR kinase. AKT1/2i selectively inhibits Akt1 and Akt2 [23], [24], while mTOR1/2i is a selective inhibitor of mTOR kinase, thus blocks the signaling of both TORC1 and TORC2 complexes [15], [25]. Surprisingly, the parental line and two independent knock-in clones displayed similar sensitivities to inhibitors in this 2-dimensional assay format (Figure 1B and Table 1). GDC-0941 EC_50_ concentrations of 0.5 µM for parental, H1047R A and H1047R B clones were utilized in subsequent experiments.

**Table 1:** Compounds used for viability and 3-D cell culture assays.

Compound ID	Specificity	Parental (EC_50_ µM)	H1047R A (EC_50_ µM)	H1047R B (EC_50_ µM)
GDC-0941	PI3K (p110α,β,δ,γ)	0.5	0.5	0.5
PI1103	PI3K (p110α,β,δ,γ), mTOR, DNA-PK	0.2	0.4	0.4
PI3Ki-A/D	PI3K (p110α,δ,γ)	1.1	2	1.6
AKT1/2i	Akt1, Akt2	>5	>5	>5
mTOR1/2i	TORC1, TORC2	2.2	1.8	1.7
Erlotinib	EGFR	4.3	>5	>5

Specificities and CellTiter-Glo viability assay EC_50_s for parental and H1047R clones.

Consistent with PI3K mutation activating the signaling pathway, the phosphorylation levels of several downstream pathway components were significantly increased in the H1047R clones compared to parental cells (Figure 1C). The phosphorylation of Akt was increased, as well as other markers of pathway activity including phospho-PRAS40, phospho-P70S6K, phospho-S6, and phospho-GSK3β. These data are consistent with a recent study showing enhancement of multiple downstream signaling effects with an endogenous PI3K mutation [12]. When the cells were treated with GDC-0941 at 0.5 µM concentrations for 1 or 4 hours, phosphorylation of all the downstream markers was reduced (Figure 1C). These included proximal pathway markers such as phospho-Akt and markers further downstream in the PI3K pathway such as phospho-GSK3β.

Augmented PTEN/PI3K pathway signaling is expected to lead to increased cell proliferation [12]. We therefore investigated the proliferative capacity of the parental and knock-in clones. When growth was measured for 48 hours, we discovered that knock-in clones proliferated approximately 2–3 fold faster than the parental cells (Figure 1D). This increase is far less than described by Isakoff et al [26] using retrovirally over-expressed PI3K in the same parental cell-type (MCF10A), nor do we see tumorigenicity in soft agar (data not shown). Our data are consistent, however, with other single oncogene knock-in phenotypes such as Ras [10], [11], where more subtle effects are observed in mutant knock-in cells compared to cells with protein over-expression. Treatment with GDC-0941 at EC_50_ and 3× EC_50_ concentrations strongly inhibited the growth of both parental and knock-in clones (Figure 1D).

To further explore the phenotypic and biological consequences of the kinase domain mutation, a microarray study was performed comparing untreated parental vs. mutant cells to those treated for 4 hours with an EC_50_ concentration of GDC-0941. Analysis of common cell cycle genes correlated with the increased proliferation we observed in the cell growth assay (Figure S1 and Table S1). Levels of cyclin D1, for example, were significantly increased in the knock-in cell line suggesting that these cells were advancing more rapidly through the G1 phase of the cell cycle. A reduction in the expression level of the cell cycle inhibitor p27^Kip1^ was observed, also suggestive of an increased proliferative capacity. GDC-0941 treatment normalized the expression of cyclin D1 and p27^Kip1^ as well as other genes differentially regulated by mutant PIK3CA (Figure S1). Altogether, these data show the PI3K kinase domain mutation drives pathway activation, increases signaling and proliferation, and GDC-0941 blocks these effects. However, there was no discernable differential ‘gene-addiction’ effect when comparing mutant versus wild-type cell growth, prompting investigation into other phenotypes for their sensitivity and selectivity to PI3K inhibitors.

### PI3K pathway activitation and epithelial-to-mesenchymal transition

**Figure 2:**
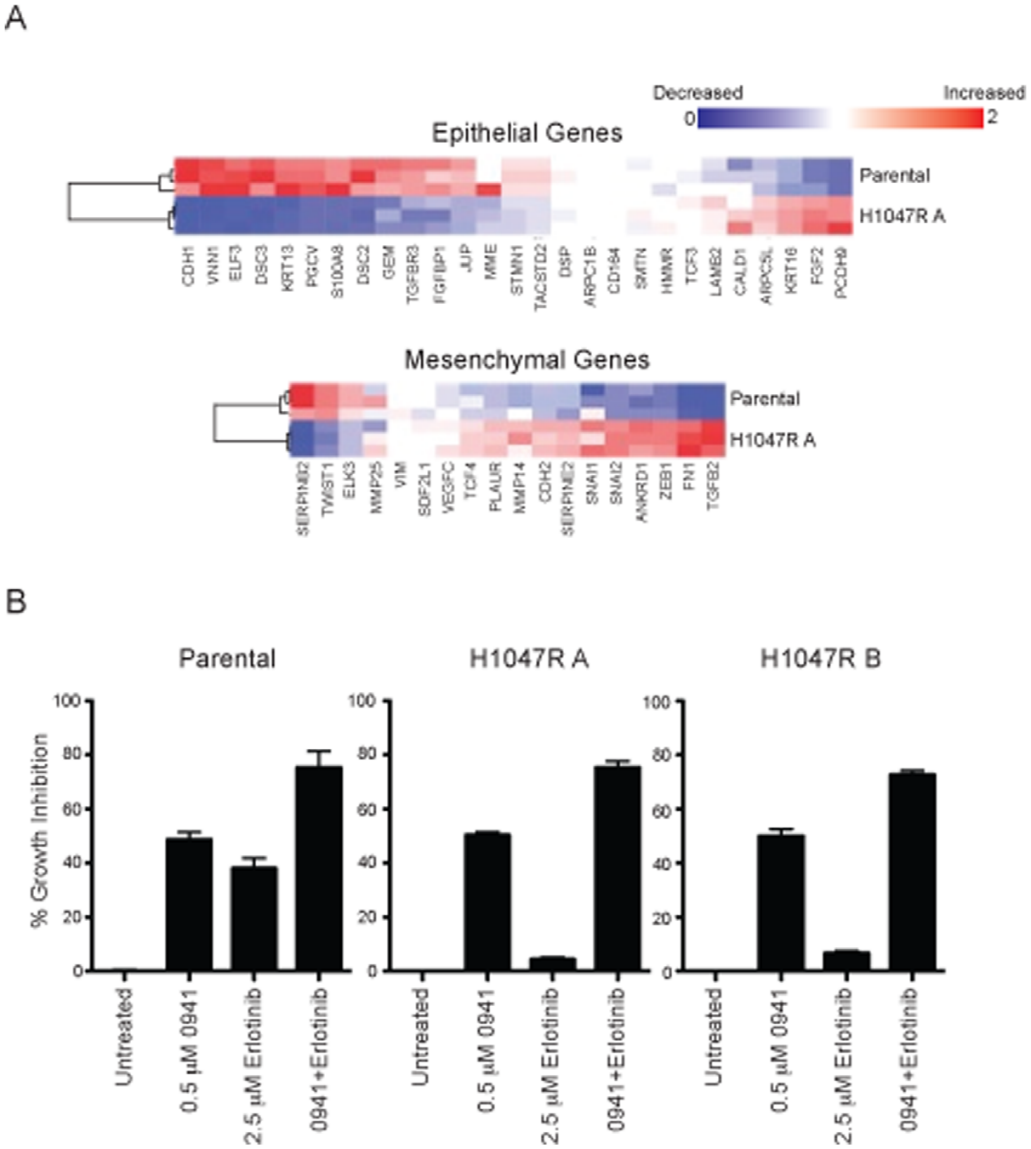
MCF10A cells undergo EMT-like changes in the presence of a PI3K mutation. (A) Comparative gene expression analysis of the parental and knock-in clone. Expression profile differences of commonly associated epithelial and mesenchymal genes by microarray are shown by heat map for a ratio of gene expression levels (H1047R A to parental). (B) Effects of GDC-0941 and erlotinib combination treatment in parental and knock-in cell lines. Percent growth inhibition data from a viability assay is shown at EC_50_ doses of single agent GDC-0941 and 2.5 µM erlotinib after drug incubation. Each bar indicates mean % inhibition ±SEM from quadruplicate wells.

MCF10A cells have a typical epithelial morphology when cultured on plastic. In contrast to the parental MCF10A cells, the knock-in clones have a spindle-like morphology more representative of fibroblasts (Figure S2). Within a tumor, these morphological changes are commonly associated with increased tumor invasiveness and metastasis. The morphological changes are also characteristic of cells that have undergone the epithelial-to-mesenchymal transition (EMT). PI3K pathway alterations have been identified as a central feature of EMT in tumor cell lines and clinical samples [27], [28], [29], [30]. For EMT to occur, complex changes in gene expression are necessary [31], [32]. Gene expression profiles were analyzed for the parental cells and the PI3K H1047R clone, and profiles associated with EMT were significantly changed. In the H1047R cells the majority of epithelial genes had decreased RNA expression compared to parental cells and conversely, the majority of the mesenchymal genes had increased expression compared to the parental cells (Figure 2A and Table S1). Through PI3K signaling, the balance between Akt1 and Akt2 rather than the overall activity of Akt has been shown to regulate the EMT response [33], [34], [35].

Resistance to EGFR inhibitors has been associated with the mesenchymal subtype [36], [37]. Consistent with this effect, the MCF10A parental cells were sensitive to the EGFR inhibitor erlotinib, while the *PIK3CA* H1047R knock-in clones were highly resistant (Figure 1B, Figure 2B, and Table 1). The resistance to erlotinib associated with the mesenchymal phenotype is also consistent with clinical data [38]. With the hypothesis that the EMT is driven through the PI3K pathway, we then tested the hypothesis that erlotinib resistance could be overcome by combining erlotinib with GDC-0941. In both parental and knock-in clones an improvement in potency was achieved when the two drugs were used in combination (Figure 2B).

### Invasiveness and the PIK3CA kinase domain mutation

Increases in invasive potential are commonly associated with EMT in preclinical models. Utilizing a microarray signature for invasiveness [39] it was found that some of the RNA expression levels were altered in the H1047R clones versus parental cells (Figure S3 and Table S1). Indeed, data from recent clinical studies found frequent PIK3CA mutations in lymph node positive tumors, suggesting increased metastasis for this genotype [40], [41], [42].

**Figure 3:**
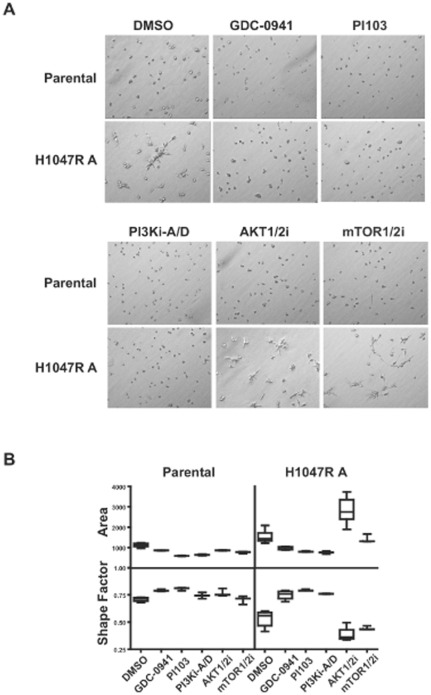
MCF10A knock-in cells show a more invasive phenotype in 3-D cell culture. (A) Parental and H1047R A cells were cultured for 2 days in the presence or absence of GDC-0941 (0.5 µM), PI103 (0.5 µM), PI3Ki-A/D (2 µM), AKT1/2i (5 µM) or mTOR1/2i (5 µM). (B) A mathematical distribution of acinar size (area) and shape (shape factor) was used to assess morphology changes with drug treatments on day 2. Data are plotted as the mean (horizontal line), middle 50% of data (box), and 95% confidence interval (lines). Pair-wise comparisons to the DMSO control were done by Student's t test. GDC-0941 and PI103 treatments resulted in significant morphology changes in both the parental and H1047R A clone (p<0.02, area or shape factor). PI3Ki-A/D treatment resulted in significant morphological changes in parental (p<0.006, area) or the H1047R clone (p<0.003, area or shape factor). Statistical significance was also achieved in the H1047R clone with the AKT1/2i (p<0.0002, area or shape factor) or mTOR1/2i (p = 0.03, shape factor).

The invasive potential of malignant cells can be reliably distinguished when cultured in the presence of a laminin-rich basement membrane [43]. Using this 3-D culture method, breast cancer cell lines have been categorized into groups based on distinct morphologies [43]. MCF10A cells form colonies of a “round” morphology with apical-basal polarity in 3-D culture, and cells with this morphology are categorized as non-invasive. When assessed in 3-D culture, the parental MCF10A colonies were round with robust cell-cell adhesion (Figure 3A). In contrast, the PI3K H1047R knock-in clones displayed a highly motile phenotype wherein individual cells grow into highly elongated projections or branches. This morphology is characteristic of the highly invasive “Stellate” group. PI3K inhibitor treatments dramatically reversed this aggressive phenotype of the H1047R knock-in clone back into a morphology resembling parental cells (Figure 3A), without impacting the viability of the acinar foci (data not shown). This overt phenotype and drug induced reversion has been confirmed in an independent source of MCF10A cells (11) that were mutated for PI3K (Figure S4).

Given that acini treated with the inhibitors displayed noticeable qualitative changes in morphogenesis, we utilized quantitative methods to more accurately assess phenotypes as a measure of drug efficacy [18]. Phase-contrast images of MCF10A parental and the knock-in clone were analyzed for total area and shape. The shape factor is a measure of morphology and yields values from 0 (flat line) to 1 (perfect circle). By these analyses, the three different PI3K inhibitors decreased the area and increased the shape factor, especially in the knock-in clone (Figure 3B). Significantly, these results indicate that increases in PTEN/PI3K pathway signaling promote an invasive phenotype, and that PI3K inhibition can reverse this effect.

In order to determine if inhibition of downstream signaling in the PI3K pathway would be effective at overcoming oncogene addiction, Akt and mTOR kinase inhibitors were assessed for effects on H1047R cells. Unexpectedly, the Akt and mTOR inhibitors, even at high concentrations of 5 µM, did not reduce the cell shape of the knock-in clone (Figure 3), suggesting morphology changes were being controlled upstream in the PI3K pathway. Interestingly, the Akt and mTOR inhibitors appeared to have the opposite effect to PI3K inhibitors, in that the Akt and mTOR inhibitors increased the invasive phenotype (Figure 3B).

We tested 10 additional breast tumor cell lines in 3-D culture in the presence or absence of PTEN/PI3K pathway inhibitors. Two of the cell lines, BT20 and MDA-MB-436, showed invasive morphologies in 3-D and were similar to the H1047R knock-in clones in response to PTEN/PI3K pathway inhibitors (Figure S5). Both of the cell lines have alterations in the PTEN/PI3K pathway. BT20 cells have the same PI3K kinase domain mutation (H1047R) as MCF10A knock-in cells, while the MDA-MB-436 cell line has lost expression of the tumor suppressor PTEN [15], [44]. Both cell lines have also been described as mesenchymal [31], [45], [46]. Thus, both increased pathway signaling and the mesenchymal subtype may be required for the invasive morphology.

**Figure 4:**
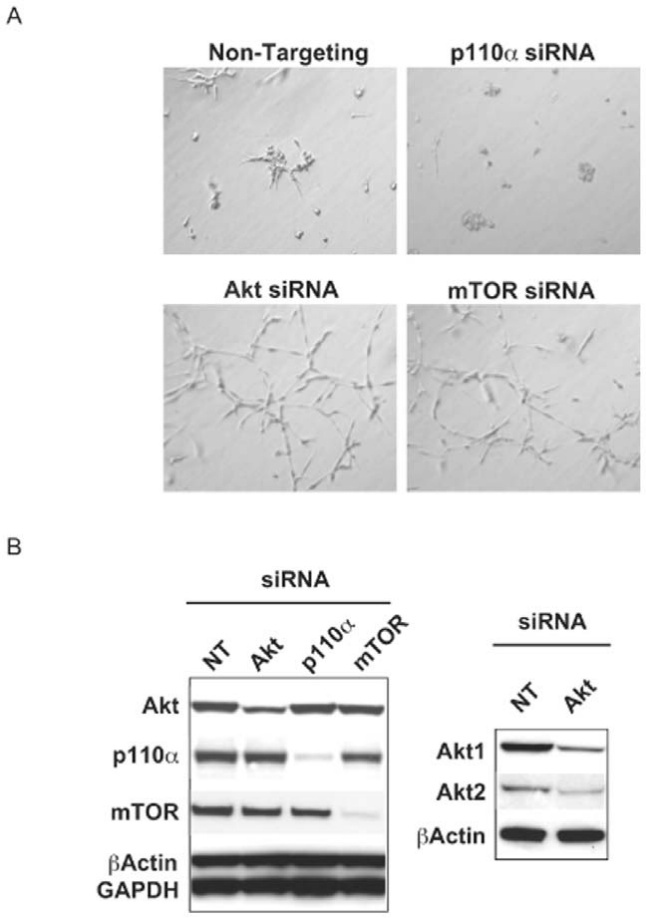
siRNA knockdown of p110α, Akt or mTOR phenocopies small molecule inhibitor treatments. (A) Knock-in cells (H1047R A) were transfected with the indicated siRNAs and plated in the 3-D culture assay for 5 days. (B) Assessment of siRNA knockdown by Western blot in the knock-in clone (H1047R A) 48 hours after transfection (NT = Non-targeting control siRNA).

To confirm morphology changes discovered using small molecule inhibitors, siRNA experiments were carried out to knock down p110α, Akt1/2, or mTOR, in the knock-in MCF10A clone. Similar to findings with small molecule inhibitors, the p110α directed siRNA clearly decreased stellate structures while knockdown of Akt or mTOR resulted in a significant promotion of invasive morphologies (Figure 4A). The effectiveness of siRNA knockdown of these targets is shown in Figure 4B. Overall, morphology changes in the parental cells with PI3K pathway inhibition were minimal in 3-D culture, likely due to decreased basal PI3K pathway activity.

**Figure 5:**
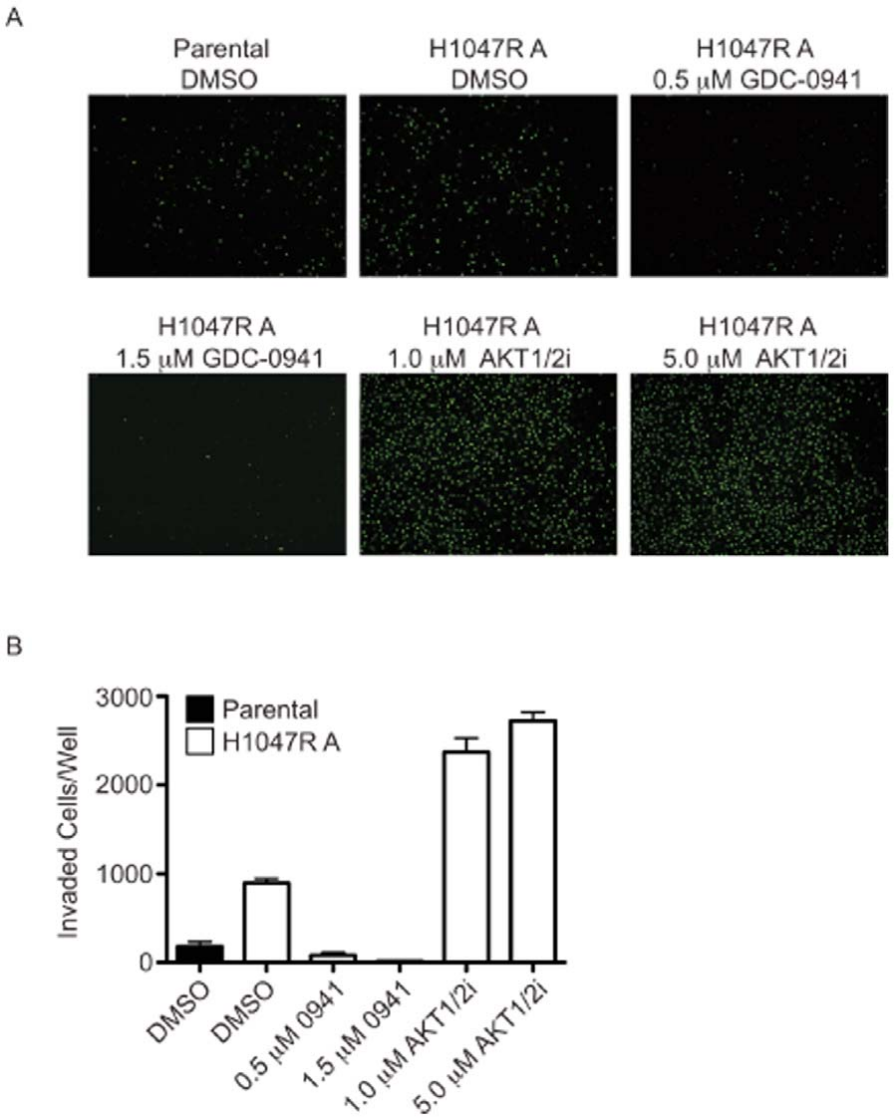
MCF10A knock-in cells show enhanced migration through matrigel. (A) Cells were plated under the conditions indicated and allowed to migrate for 24 hours. Representative images for each condition are shown. (B) Quantification of migrated cells in triplicate wells.

The process of tumor metastasis and invasion involves the migration of individual cells from the primary tumor through a basement membrane. The cells then enter the bloodstream or lymphatic vessels and ultimately seed into a distant organ site [47]. We investigated motility of the MCF10A isogenic cells through matrigel and found that the H1047R knock-in clone migrated approximately 5-fold faster than the parental clones over 24 hours (Figure 5). In the migration assay, treatments with inhibitors also showed a similar trend to the phenotypes observed in matrigel. GDC-0941 decreased the number of migrated knock-in cells, while AKT1/2i increased the number of migrated knock-in cells. Effects of GDC-0941 and AKT1/2i were not significant for the parental cells in the migration assay (data not shown). Thus, the morphologies observed in 3-D culture are due, at least in part, to increased cell motility.

**Figure 6:**
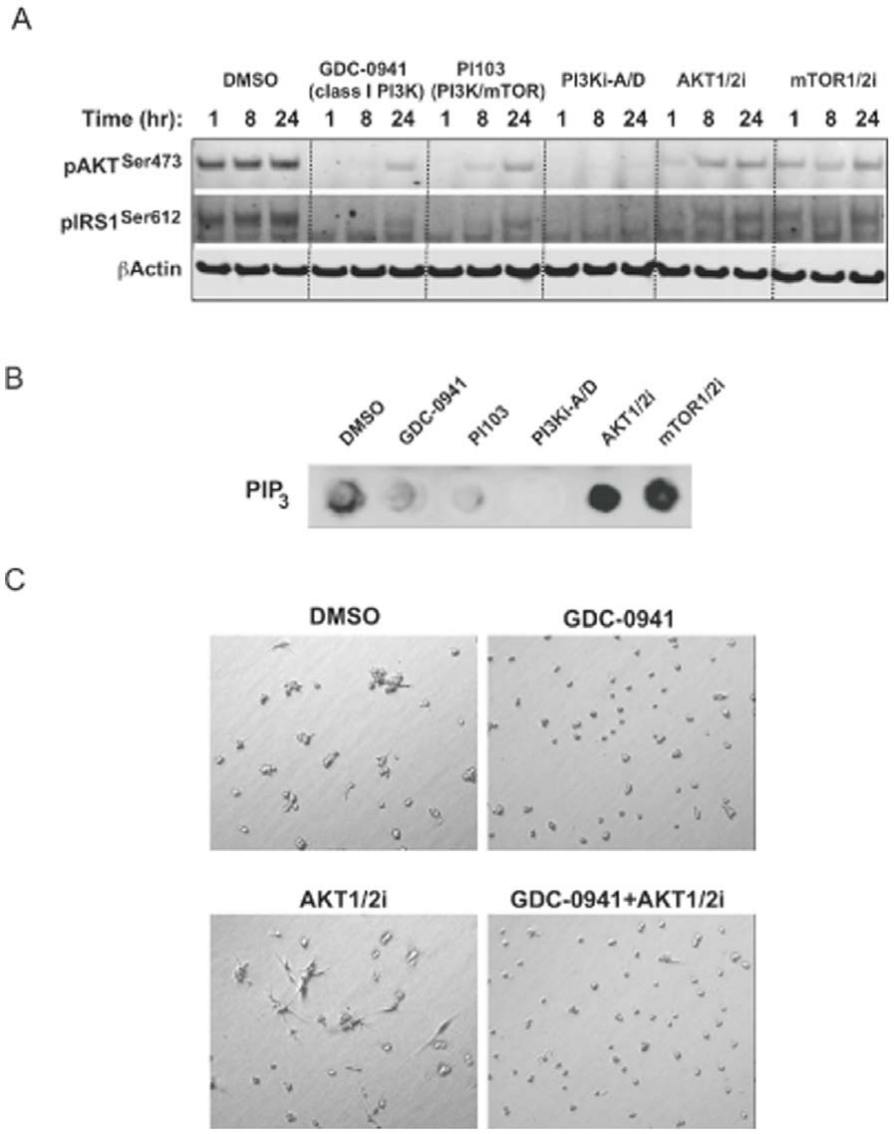
PI3K signaling responses to PI3K pathway inhibition. (A) Knock-in cells (H1047R A) were cultured with inhibitors at concentrations used in the 3-D culture assay and analyzed by Western blotting for indicated treatment times. (B) PIP_3_ levels assessed at 48 hours post treatment with 3-D culture concentrations of inhibitors in the knock-in clone (H1047R A). (C) PI3K and Akt inhibitor effects can be distinguished in 3-D culture through variations in cellular morphology. Phase contrast images of the knock-in clone (H1047R A) show representative phenotypes of treatment with 0.5 µM GDC-0941, 5 µM AKT1/2i, or the same compound doses in combination after 2 days in culture.

The effect of compounds on downstream PI3K pathway markers was investigated in the H1047R knock-in clone at the same drug concentrations and media conditions used in the 3-D culture assay (Figure 6A). The PI3K pathway inhibitors all showed similar inhibition of phospho-Akt^Ser473^ and phospho-IRS1^Ser612^. As expected, amounts of PIP_3_ decreased with PI3K inhibitor treatments (Figure 6B). In contrast, cellular PIP_3_ levels were elevated above basal levels upon treatment with either an Akt or mTOR kinase inhibitor. Taken together, these data support the hypothesis that PI3K pathway blockade promotes feedback to PIP_3_, but if PI3K itself is not inhibited, PIP_3_ levels can accumulate and promote the invasive morphology. In support of this hypothesis, when GDC-0941 was combined with AKT1/2i in 3-D culture, the morphology resembled GDC-0941 treatment alone (Figure 6C). Significantly, these findings suggest a potential therapeutic difference between PI3K and Akt inhibitors in their effectiveness at controlling invasion and metastasis. We are currently investigating the signaling mediators of these differences.

When cultured on plastic (2-D), the H1047R knock-in clones displayed increased PI3K pathway activity and proliferation, but oncogene addiction was not observed. We found that the H1047R mutation was linked to EMT by analysis of epidermal and mesenchymal gene signatures, rescue of EGFR inhibitor resistance by a PI3K inhibitor, and acinar morphologies in 3-D culture. PI3K inhibitors and siRNA reversed the morphologies in 3-D culture, but mTOR and Akt inhibitors and siRNA worsened the invasive phenotype. This was due to increased feedback to PIP_3_ that cannot be controlled by inhibitors downstream of PI3K.

These data suggest that invasion may be the most relevant phenotype to assess for PI3K oncogene addiction. Consistent with this hypothesis is a previous in vivo study that showed knocking-out mutant PIK3CA in a fully tumorigenic colon cancer cell line had more pronounced effects on metastasis than on primary tumor growth [48]. Not only do these results demonstrate invasion is a core phenotype of mutant PI3K, but that only direct PI3K-inhibitors are effective in reversing it, and targeting downstream nodes in the pathway can in fact promote the invasive phenotype.

In summary, the H1047R mutant of PIK3CA increases PTEN/PI3K pathway signaling and markers of EMT and cell migration phenotypes in isogenic breast epithelial cell lines. Notably, pathway targeted agents dramatically diverge in their ability to revert these phenotypes, with direct PI3K inhibitors representing the optimal intervention node for mutant PI3K cancers. These data suggest that invasion-based readouts and biomarkers may be the most appropriate end points to reveal the ‘gene-addiction’ responses to PI3K inhibitors in the clinic.

## Supporting Information

Figure S1 **Differential mRNA expression of cell cycle genes in triplicate microarray samples.** Error bars indicate ±SEM.
(TIF)

Figure S2 **Phase-contrast images of the parental and knock-in clones in culture.**
(TIF)

Figure S3 **MCF10A cells with a PI3K mutation show gene expression patterns associated with invasiveness.** Comparative gene expression analysis of the MCF10A parental and H1047R clone A. Microarray differences presented as a heat map are indicated as ratios (H1047R A to parental) for genes commonly linked to invasiveness.
(TIF)

Figure S4 **Horizon MCF10A H1047R knock-in cells show a more invasive phenotype in 3-D cell culture.** Parental and H1047R knockin clones were cultured for 24 hours in Geltrex Reduced Growth Factor Basement Membrane Matrix (Invitrogen) in the presence or absence of GDC-0941 (1 µM). This type of matrigel was used to augment the invasive morphologies of the H1047R knockin clones.
(TIF)

Figure S5 **GDC-0941 inhibits invasive morphologies in breast tumor cell lines.** (A) BT20 cells were cultured for 24 hours in the presence or absence of inhibitors at EC_50_ viability concentrations (GDC-0941 (0.6 µM), PI103 (0.4 µM), PI3Ki-A/D (1.2 µM), AKT1/2i (3 µM), or mTOR1/2i (2 µM)). (B) MDA-MB-436 cells were cultured for 48 hours in the presence or absence of inhibitors at EC_50_ viability concentrations (GDC-0941 (0.8 µM), PI103 (0.5 µM), PI3Ki-A/D (1.5 µM), AKT1/2i (3.8 µM), or mTOR1/2i (1.7 µM)).
(TIF)

Table S1 **Gene probe sets utilized for microarray analysis.**
(DOC)
