# Supplementary figures and images for: Active PI3K Pathway Causes an Invasive Phenotype Which Can Be Reversed or Promoted by Blocking the Pathway at Divergent Nodes
Source: PLoS One. 2012 May 3;7(5):e36402. doi: 10.1371/journal.pone.0036402 (PMC3343052; doi:10.1371/journal.pone.0036402)

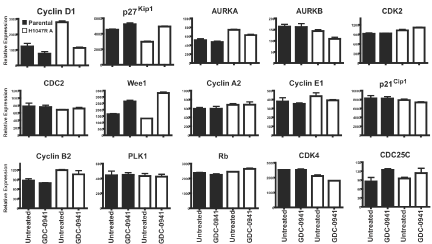

Supplement: Figure S1 — Differential mRNA expression of cell cycle genes in triplicate microarray samples. Error bars indicate ±SEM. (TIF) [file pone.0036402.s001.tif]

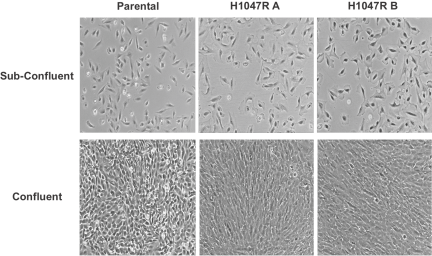

Supplement: Figure S2 — Phase-contrast images of the parental and knock-in clones in culture. (TIF) [file pone.0036402.s002.tif]

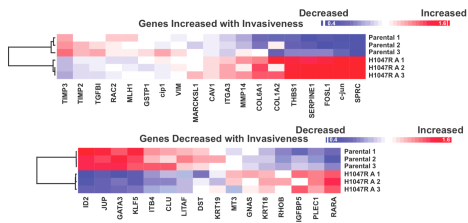

Supplement: Figure S3 — MCF10A cells with a PI3K mutation show gene expression patterns associated with invasiveness. Comparative gene expression analysis of the MCF10A parental and H1047R clone A. Microarray differences presented as a heat map are indicated as ratios (H1047R A to parental) for genes commonly linked to invasiveness. (TIF) [file pone.0036402.s003.tif]

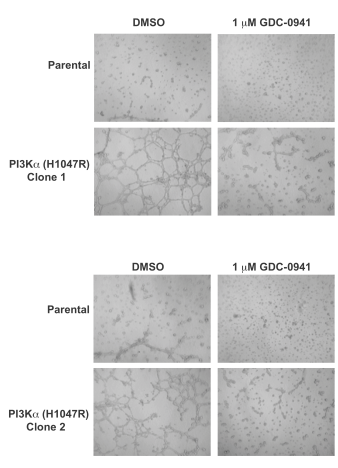

Supplement: Figure S4 — Horizon MCF10A H1047R knock-in cells show a more invasive phenotype in 3-D cell culture. Parental and H1047R knockin clones were cultured for 24 hours in Geltrex Reduced Growth Factor Basement Membrane Matrix (Invitrogen) in the presence or absence of GDC-0941 (1 µM). This type of matrigel was used to augment the invasive morphologies of the H1047R knockin clones. (TIF) [file pone.0036402.s004.tif]

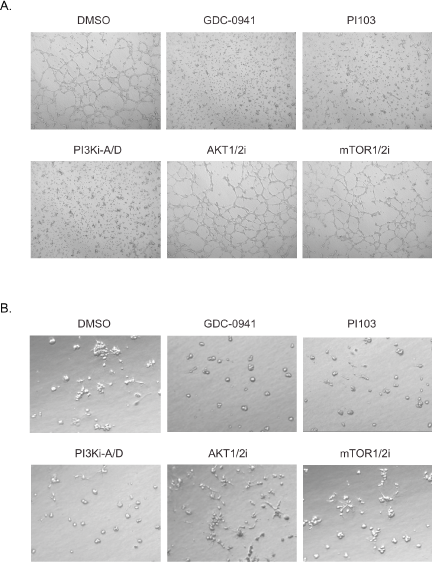

Supplement: Figure S5 — GDC-0941 inhibits invasive morphologies in breast tumor cell lines. (A) BT20 cells were cultured for 24 hours in the presence or absence of inhibitors at EC50 viability concentrations (GDC-0941 (0.6 µM), PI103 (0.4 µM), PI3Ki-A/D (1.2 µM), AKT1/2i (3 µM), or mTOR1/2i (2 µM)). (B) MDA-MB-436 cells were cultured for 48 hours in the presence or absence of inhibitors at EC50 viability concentrations (GDC-0941 (0.8 µM), PI103 (0.5 µM), PI3Ki-A/D (1.5 µM), AKT1/2i (3.8 µM), or mTOR1/2i (1.7 µM)). (TIF) [file pone.0036402.s005.tif]
