# Supplementary material for: Active PI3K Pathway Causes an Invasive Phenotype Which Can Be Reversed or Promoted by Blocking the Pathway at Divergent Nodes
Source: PLoS One. 2012 May 3;7(5):e36402. doi: 10.1371/journal.pone.0036402 (PMC3343052; doi:10.1371/journal.pone.0036402)
Supplement: Table S1 — Gene probe sets utilized for microarray analysis. (DOC) [file pone.0036402.s006.doc]

Table S1.

| **Cell Cycle** |  |  |  |
| --- | --- | --- | --- |
| Probe ID | Gene | P-value | Ratio (H1047R to Parental) |
| 209112_at | Kip1 (p27) | 2.67E-04 | 0.65 |
| 212533_at | WEE1 | 3.43E-02 | 0.80 |
| 202246_s_at | CDK4 | 3.36E-02 | 0.84 |
| 203213_at | CDC2 | 3.62E-01 | 0.88 |
| 209464_at | AURKB | 1.92E-01 | 0.88 |
| 202284_s_at | Cip1 (p21) | 5.89E-01 | 0.95 |
| 1555900_at | PLK1 | 8.85E-01 | 0.98 |
| 203132_at | RB1 | 2.64E-01 | 1.03 |
| 204252_at | CDK2 | 8.61E-02 | 1.12 |
| 205167_s_at | CDC25C | 5.45E-01 | 1.12 |
| 213226_at | CCNA2 | 1.33E-01 | 1.14 |
| 213523_at | CCNE1 | 3.59E-01 | 1.16 |
| 214710_s_at | CCNB2 | 2.05E-02 | 1.38 |
| 204092_s_at | AURKA | 1.14E-02 | 1.45 |
| 208712_at | CCND1 | 8.13E-03 | 2.29 |

| **Mesenchymal** |  |  |  |
| --- | --- | --- | --- |
| Probe ID | Gene | P-value | Ratio (H1047R to Parental) |
| 204614_at | SERPINB2 | 1.52E-02 | 0.05 |
| 213943_at | TWIST1 | 2.33E-05 | 0.35 |
| 221773_at | ELK3 | 8.00E-03 | 0.58 |
| 207289_at | MMP25 | 8.00E-01 | 0.93 |
| 201426_s_at | VIM | 9.85E-01 | 1.00 |
| 218681_s_at | SDF2L1 | 6.51E-02 | 1.15 |
| 209946_at | VEGFC | 2.22E-02 | 1.22 |
| 203753_at | TCF4 | 1.36E-02 | 1.51 |
| 210845_s_at | PLAUR | 5.88E-04 | 1.67 |
| 202827_s_at | MMP14 | 2.92E-02 | 1.71 |
| 203440_at | CDH2 | 3.88E-04 | 1.72 |
| 212190_at | SERPINE2 | 2.38E-05 | 2.01 |
| 219480_at | SNAI1 | 8.93E-02 | 2.30 |
| 213139_at | SNAI2 | 1.57E-06 | 2.83 |
| 206029_at | ANKRD1 | 2.78E-04 | 2.91 |
| 21085_s_at | ZEB1 | 2.50E-05 | 2.92 |
| 214702_at | FN1 | 5.16E-03 | 7.53 |
| 220407_s_at | TGFB2 | 1.06E-03 | 7.99 |

Table S1 (continued).

| **Epithelial** |  |  |  |
| --- | --- | --- | --- |
| Probe ID | Gene | P-value | Ratio (H1047R to Parental) |
| 201131_s_at | CDH1 | 1.21E-02 | 0.00 |
| 205844_at | VNN1 | 8.47E-04 | 0.03 |
| 210827_s_at | ELF3 | 3.06E-03 | 0.05 |
| 206032_at | DSC3 | 1.28E-02 | 0.06 |
| 207935_s_at | KRT13 | 1.26E-02 | 0.09 |
| 204620_s_at | PGCV | 3.61E-04 | 0.16 |
| 202917_s_at | S100A8 | 1.67E-02 | 0.15 |
| 226817_at | DSC2 | 1.11E-02 | 0.15 |
| 204472_at | GEM | 1.76E-03 | 0.29 |
| 204731_at | TGFBR3 | 2.39E-04 | 0.30 |
| 205014_at | FGFBP1 | 3.79E-03 | 0.37 |
| 201015_s_at | JUP | 1.08E-04 | 0.42 |
| 203434_s_at | MME | 1.34E-01 | 0.50 |
| 200783_s_at | STMN1 | 1.16E-04 | 0.64 |
| 202286_s_at | TACSTD2 | 3.09E-03 | 0.68 |
| 200606_at | DSP | 1.76E-02 | 0.88 |
| 201954_at | ARPC1B | 7.52E-02 | 0.95 |
| 208405_s_at | CD164 | 4.78E-01 | 0.97 |
| 1556030_at | SMTN | 6.07E-01 | 1.05 |
| 207165_at | HMMR | 3.77E-01 | 1.08 |
| 209153_s_at | TCF3 | 5.18E-02 | 1.09 |
| 216264_s_at | LAMB2 | 2.56E-02 | 1.38 |
| 243084_at | CALD1 | 8.26E-02 | 1.75 |
| 226915_s_at | ARPC5L | 5.42E-05 | 1.82 |
| 209800_at | KRT16 | 4.30E-05 | 2.77 |
| 204422_s_at | FGF2 | 2.44E-04 | 3.87 |
| 219737_s_at | PCDH9 | 1.03E-02 | 5.72 |

Table S1 (continued).

| **Genes Increased with Invasiveness** | | |  |
| --- | --- | --- | --- |
| Probe ID | Gene | P-value | Ratio (H1047R to Parental) |
| 201150_s_at | TIMP3 | 7.35E-04 | 0.65 |
| 224560_at | TIMP2 | 3.36E-02 | 0.83 |
| 201506_at | TGFBI | 6.79E-02 | 0.86 |
| 213603_s_at | RAC2 | 2.06E-02 | 0.88 |
| 202520_s_at | MLH1 | 1.20E-01 | 0.91 |
| 200824_at | GSTP1 | 2.00E-01 | 0.94 |
| 202284_s_at | Cip1 (p21) | 5.89E-01 | 0.95 |
| 201426_s_at | VIM | 9.85E-01 | 1.00 |
| 200644_at | MARCKSL1 | 1.64E-01 | 1.11 |
| 212097_at | CAV1 | 2.28E-03 | 1.13 |
| 201474_s_at | ITGA3 | 1.05E-03 | 1.31 |
| 160020_at | MMP14 | 7.29E-02 | 1.32 |
| 212940_at | COL6A1 | 5.77E-02 | 1.74 |
| 202404_s_at | COL1A2 | 6.33E-02 | 1.84 |
| 235086_at | THBS1 | 1.40E-04 | 2.47 |
| 1568765_at | SERPINE1 | 1.58E-03 | 3.05 |
| 204420_at | FOSL1 | 1.69E-03 | 3.23 |
| 201466_s_at | c-jun | 8.48E-05 | 3.43 |
| 212667_at | SPRC | 9.11E-07 | 4.52 |

| **Genes Decreased with Invasiveness** | | |  |
| --- | --- | --- | --- |
| Probe ID | Gene | P-value | Ratio (H1047R to Parental) |
| 213931_at | ID2 | 1.78E-03 | 0.35 |
| 201015_s_at | JUP | 1.08E-04 | 0.42 |
| 209602_s_at | GATA3 | 1.00E-03 | 0.43 |
| 209211_at | KLF5 | 1.15E-04 | 0.44 |
| 204990_s_at | ITB4 | 8.00E-04 | 0.59 |
| 208792_s_at | CLU | 1.54E-02 | 0.66 |
| 200704_at | LITAF | 1.00E-02 | 0.69 |
| 1560296_at | DST | 9.34E-02 | 0.76 |
| 201650_at | KRT19 | 2.40E-01 | 0.89 |
| 205970_at | MT3 | 6.74E-01 | 1.05 |
| 228173_at | GNAS | 9.67E-02 | 1.17 |
| 201596_x_at | KRT18 | 5.06E-04 | 1.35 |
| 212099_at | RHOB | 4.40E-02 | 1.40 |
| 211958_at | IGFBP5 | 2.75E-02 | 1.58 |
| 201373_at | PLEC1 | 7.19E-04 | 1.62 |
| 203749_s_at | RARA | 1.38E-03 | 2.11 |
